# Secretary’s Report

**Published:** 1894-11

**Authors:** T. J. Turner

**Affiliations:** Philadelphia, Pa.


### AN ACT

### TO FIX THE PAY, ALLOWANCES, PENSIONS, RETIREMENT AND RANK OF THE VETERINARIANS OF THE UNITED STATES ARMY.

Be it enacted by the Senate and House of Representatives of the United States of America in Congress assembled:

Section 1. That the United States Army Veterinarians shall have the pay, allowances, pensions, retirements, tenure of office, and the relative rank of Second Lieutenant of Cavalry; their appointment to date from original entry into the Army. Provided, however, that hereafter candidates for the United States Army Veterinary service shall comply with all the preliminaries now required from candidates for "The Army Medical Corps," and shall pass such examinations as the Secretary of War may direct.

Sec. 2. This Act shall be in force from and after its approval.

Sec. 3. All Acts inconsistent with this Act are hereby repealed.

### Secretary's Report, United States Veterinary Medical Association.

Philadelphia, Pa., Sept., 1894.

Mr. President and Gentlemen: While electing officers at the last annual meeting at Chicago, your honorable body, from compassion (I having a strained knee, and as officers are frequently selected from such like misfortunes) I am sure and not from any fitness for the position, chose me as your Secretary for the ensuing year.

Many were the apprehensions which possessed me as the result of the ballot was read. But I resolved not to shirk my duty nor shrink from any task your honorable body might deem it wise to impose. For the great honor conferred I felt humbly proud and my heart swelled within me as by degrees I realized the trust and confidence thus bestowed. I shall not by words endeavor to express to you my appreciation of so great a distinction, but rather try by actions earnest and work zealous to the best and fullest extent of my ability for the good of the Association, to in a measure make you not regret your trust imposed. Much if not all the work in connection with this office was entirely new to me, and if at times I have made mistakes and been apparently derelict, to confusion and ignorance attribute it, rather than to lack of anxiety and earnest desire to do all possible for the welfare of the organization.

Notifications of their election at last meeting to all foreign honorary members were sent out and responses received. The translation of these letters were in substance all the same, viz., thanking the Association for the honor conferred and accepting the same with thanks. During the year about 400 communications have been received at this office and about 2,600 have been sent out.

The latter including letters, notices of indebtedness twice a year, and to those in arrears past the expulsion period a circular letter was written to try, if possible, to obtain some recognition of the receipt of the notice and liability to and intention to pay indebtedness to the Association. This was productive of good, as from some money came, and from others letters of promise and explanation. Lists of committees and officers were also sent, together with postals to each in regard to proceedings, and programs with about 600 invitations to members of various State organizations, State Sanitary Live Stock Boards, etc., as well as the members of various faculties of the veterinary colleges of the United States.

There have been received sixteen applications for membership this year and one for honorary membership, and although the business stress has been severe and electricity has done away vastly with the necessity for a certain class of horse stock the veterinary profession prospers as well as do others. In notifying applicants for membership of the disposition made of their application, some very rare and interesting letters have been received and are on file, as are also some from resident State secretaries. When trying to interest some professional brethren to become interested in the national work, we have even been dubbed aristocrats and many other high flown names.

There have been sent out 140 cloth bound copies of the proceedings of 1893 and 286 paper bound copies. We have now 322 regular qualified members and twenty-six honorary members. Out of this number only sixty-six are in arrears to the extent of expulsion. Certificates have been issued to twenty during this year.

Since my occupancy of this office there has been received up to July 1st, $295.25, disbursed, $119.40. Balance on hand and forwarded treasurer, as per constitution and by-laws, $175.85. Since that there has been received $280.25 and disbursed $195.00, leaving balance on hand, September 14th, $85.00. With the ever increasing expense of our now most healthy and prosperous organization some changes become not only necessary but mandatory. Every member now wishes a copy of the proceedings, and when it is known that the cost of printing the same is $2.00 it is easily seen that with the annual dues only $2.00 the Association has but little if any means for current expenses, such as stationery, etc. Something must be done in regard to this matter and no doubt will be by the Association under the suggestions from the Comitia Minora and other committees. We have no time to defer this matter, but must act upon it in some manner at this meeting.

Several delegates are with us from several States and inter-State organizations, and it is with much pleasure we note them, as it is indicative of increasing interest.

In conclusion, co-workers, in a common cause, I wish to thank you for your kindly aid and assistance in helping me forward to so successful termination in the work of this office, and especially do I wish to thank our former Secretary and honorable President for his inestimable assistance to me in this work.

Respectfully,

T. J. Turner.

